# Artificial Intelligence in Detecting Periodontal Disease From Intraoral Photographs: A Systematic Review

**DOI:** 10.1016/j.identj.2025.100883

**Published:** 2025-07-09

**Authors:** Kaijing Mao, Khaing Myat Thu, Kuo Feng Hung, Ollie Yiru Yu, Richard Tai-Chiu Hsung, Walter Yu-Hang Lam

**Affiliations:** a Faculty of Dentistry, The University of Hong Kong, Hong Kong Special Administrative Region, China; b Department of Computer Science, Hong Kong Chu Hai College, Hong Kong Special Administrative Region, China

**Keywords:** Artificial intelligence, Machine learning, Periodontal disease, Photograph, Gum disease, Gingivitis

## Abstract

This systematic review aims to evaluate the methodological characteristics and clinical performance of artificial intelligence (AI) models in detecting periodontal disease using digital intraoral photographs. This review includes peer-reviewed publications and conference proceedings in English, focusing on clinical studies of human periodontal diseases. Intraoral photographs served as the primary data source, with fluorescent and microscopic dental images excluded. The methodological characteristics and performance metrics of clinical studies reporting on AI models were analysed. Twenty-six studies met the review criteria. Various image acquisition devices were used by the resarchers including professional cameras, intraoral cameras, smartphones, and home-use devices. Ten studies used clinical examinations as reference methods, while 16 used visual examinations. Eight studies involved multiple experts in dataset annotation. Only 9 studies employed multiple intraoral views for their AI models, with the remaining studies focusing solely on the frontal view. Regarding AI tasks, 17 studies used classification, 4 used detection, and 5 used segmentations. Performance metrics varied widely and were assessed at multiple levels. Classification studies showed accuracies ranging from 0.46 to 1.00, detection studies showed accuracies from 0.56 to 0.78, and segmentation studies achieved Intersection over Union (IoU) scores of 0.43 to 0.70. AI models show potential for detecting periodontal disease from intraoral photographs, but their clinical use faces challenges. Future research should focus on improving reporting standards, standardising evaluation metrics, performing external tests, enhancing data quality, and using clinical gold standards as reference methods. Furthermore, efforts should focus on promoting transparency, integrating ethical considerations, minimising misclassification, and advancing the development of explainable and user-friendly AI systems to enhance their clinical applicability and reliability.

## Introduction

Periodontal disease affects the tissues surrounding and supporting the teeth. In its mild form, gingivitis is characterised by symptoms such as red, swollen, and bleeding gingiva.^1^ In its more severe form, periodontitis leads to the progressive loss of supporting tissues, including gingiva and bone, resulting in loose teeth and potential tooth loss.^2^ Severe periodontal diseases are estimated to impact approximately 19% of the global adult population, totaling over 1 billion cases worldwide.^3^ Although largely preventable through self-care measures like tooth brushing and interdental cleaning, untreated periodontal disease remains a significant health burden.^3^, ^4^, ^5^

Early detection of periodontal diseases is crucial for effective prevention and early management of this disease. Clinical and radiographic examination remains the gold standard for diagnosing, determining prognosis, and planning treatment for periodontal disease,^6^^,^^7^ many low- and middle-income countries lack sufficient services to prevent and treat oral health conditions.^8^ Detecting periodontal disease from intraoral photographs may offer a faster option for early detection. Clinical photography has become increasingly important in patient management and has demonstrated clinically acceptable accuracy compared with visual clinical examination.^9^ Digital intraoral photographs captured by professional single-lens reflex (SLR) cameras are used for monitoring patients' periodontal health, including gingival recession.^10^ Additionally, some clinicians use intraoral cameras to capture detailed images of the oral cavity, enhancing clinical diagnosis and patient communications.^11^ Recently, the widespread availability of smartphones has made digital intraoral photographs more convenient. Smartphones, with their high-resolution imaging capabilities, are comparable to professional cameras in terms of picture quality.^12^, ^13^, ^14^ Given their ease of use, radiation-free nature, and connectivity to the internet, digital intraoral photographs are gaining popularity in teledentistry, which involves the remote provision of dental consultation and oral health education through mobile technology rather than direct face-to-face patient contact.^15^, ^16^, ^17^ Digital intraoral photographs have the potential to facilitate earlier diagnosis of periodontal disease by prompting patients to seek a dental professional for a proper diagnosis. However, this is only possible if the photographs have satisfactory accuracy compared to clinical and radiographic examinations.

The integration of artificial intelligence (AI) into healthcare has significantly expanded the applications of dental imaging,^18^^,^^19^ particularly for detecting periodontal disease across various imaging modalities.^20^ Moreover, when evaluating the outcomes of AI applications in dental imaging, it is important to understand the specific tasks of individual AI models, including image classification, detection, and segmentation.^21^ Image classification involves categorising an entire image, typically in a binary format, such as indicating the presence or absence of disease. Detection and segmentation involve distinguishing the area of interest from the rest of the image. Detection involves marking the area of interest with bounding boxes, that is, localisation, while segmentation provides an exact outline of the area of interest using pixel-wise annotations.

For the clinical application of AI models, their performance must be rigorously evaluated using various datasets. Typically, 3 subsets of datasets are employed for the development and evaluation of AI models: the training set, the validation set, and the test set.^22^^,^^23^ The training set is used to train the model, enabling it to learn patterns and relationships within the data. The validation set, on the other hand, is utilised during the model development stage to fine-tune the model’s parameters and prevent overfitting. The test set serves as the gold standard for the final evaluation of the model and is only used once the model has been fully developed using the training and validation sets. One commonly used method for validation is k-fold cross-validation. In this technique, the dataset is divided into K equal subsets, and the model is trained and validated K times. Each time, a different subset serves as the validation set while the remaining subsets form the training set. This approach ensures robust performance evaluation and helps reduce overfitting.^24^^,^^25^

While the use of AI for detecting periodontal disease using image modalities such as radiographic and fluorescent images has been reported,^20^ there is currently a lack of comprehensive reviews summarising the use of photographs and AI in periodontal disease detection. This review aims to address this gap by evaluating studies that utilise intraoral photographs to detect periodontal disease. It seeks to compare the clinical performance of AI models against reference methods in terms of accuracy, hypothesising that AI models using intraoral photographs achieve accuracy comparable to (80% or above) that of reference methods. Additionally, this review examines the methodological characteristics of AI studies in periodontal disease detection to provide insights into their strengths and limitations.

## Materials and methods

### Protocols

This systematic review was conducted according to the Preferred Reporting Items for Systematic Reviews and Meta-Analyses (PRISMA) statement guidelines.^26^ The study protocol was registered with PROSPERO (CRD42024561644).

### Eligibility criteria

The identified records were screened and evaluated based on the following inclusion criteria:

1. The article must be an original peer-reviewed publication or conference proceedings written in English.
2. The study is conducted *in vivo* and focuses on human periodontal diseases.
3. The data source consists of intraoral photographs, excluding fluorescent and microscopic dental images.
4. The study reports the use of AI models for detecting periodontal diseases and includes the performance metrics of these AI models.

Records were excluded based on the following exclusion criteria:

1. Studies involving animals.
2. Studies that used data sources other than intraoral photographs.

### PICO question

How does the clinical performance of AI models in detecting periodontal disease from intraoral photographs compare to reference methods in terms of accuracy?

Participants: Subjects with digital intraoral photographs showing teeth and gingiva.

Intervention: The use of AI models to detect periodontal disease.

Comparison: Reference methods such as clinical dental examinations or visual judgment by dental professionals, and subsequent image annotations by a manual or semi-automatic method.

Outcomes: Performance metrics, including accuracy, sensitivity, specificity, and Intersection over Union (IoU).

### Search strategy

Four electronic databases (PubMed, MEDLINE, Scopus, and Web of Science) were searched to identify publications meeting the inclusion criteria. The search will be conducted until January 30, 2025. Each database was searched using specific keywords, as shown in Table 1. Supplementary searches were conducted on Google Scholar, and reference lists from selected studies were reviewed to identify any potentially missed studies.

**Table 1:** The keywords of an electronic search in various databases.

Database	Keywords
PubMed &	("Gingivitis"[MeSH] OR "Periodontal Diseases"[MeSH] OR "Gum disease" OR "Periodontitis"[MeSH]) AND ("Artificial Intelligence"[MeSH] OR "Deep Learning"[MeSH] OR "Machine Learning"[MeSH] OR "AI-based" OR "Computer Vision").
MEDLINE	
Scopus &	("Gingivitis" OR "Periodontal Diseases" OR "Gum disease" OR "Periodontitis") AND ("Artificial Intelligence" OR "Deep Learning" OR "Machine Learning" OR "AI-based" OR "Computer Vision").
Web of Science	

### Study selection

Two assessors (K.M. and K.M.T.) independently conducted a 2-stage screening process: first, by screening the titles and abstracts, and then by reading the full texts. Data were managed using Covidence systematic review software (Veritas Health Innovation). Duplicates were removed both within and across databases. The full texts of potentially relevant articles were retrieved and assessed based on the eligibility criteria. Any disagreements regarding the selection of studies were discussed and resolved.

### Data extraction

For the studies that met the eligibility criteria, the following information was extracted using a pre-designed data extraction form: title, authors' names, year of publication, location of the study, image acquisition device, sample size, dataset information, type of periodontal disease, disease location, reference method, type of AI task (classification, detection, segmentation), AI model information, sample allocation (training, validation, test), and performance measures. Data were extracted from the included studies by one assessor (K.M.), revised by a second assessor (K.M.T.), and verified by a third assessor (W.Y.H.L.).

### Data analysis

A meta-analysis was not possible due to significant methodological and clinical heterogeneity among the included studies. Instead, performance metrics were grouped according to the type of AI task that is, classification, detection, and segmentation.

### Risk of bias and applicability

Two assessors (K.M. and K.M.T) independently assessed study quality using the Quality Assessment of Diagnostic Accuracy Studies (QUADAS-2) criteria, compared results, and resolved discrepancies through discussion. If disagreements persisted, a third reviewer (W.Y.H.L.) provided the final decision. The QUADAS-2 evaluates 4 domains: patient selection, index test, reference method, and flow and timing. Each domain was rated as having a low risk, high risk, or unclear risk of bias according to pre-defined criteria.

## Results

### Study selection

**Fig. 1:**
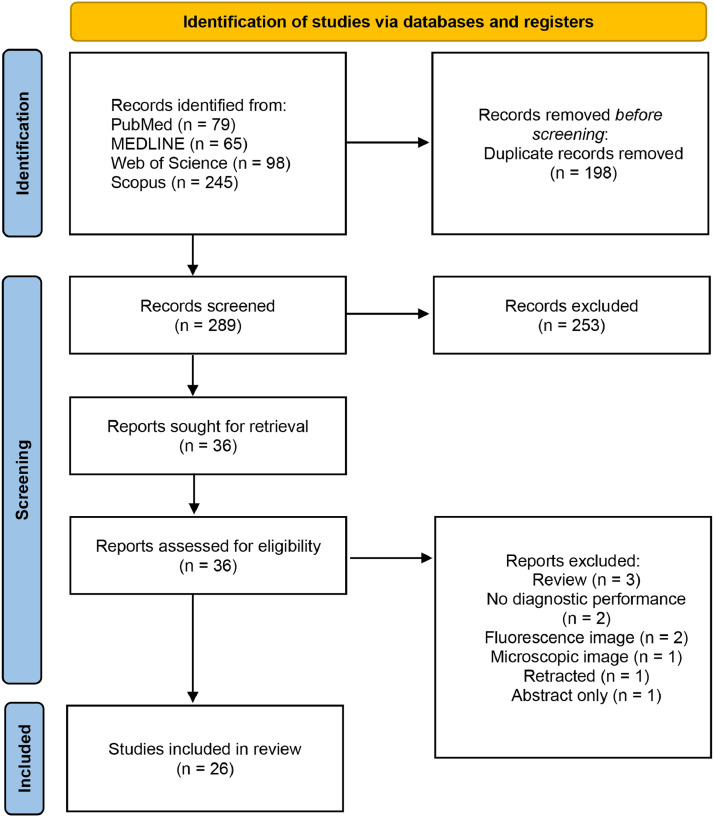
PRISMA flow diagram illustrating the search process.

The systematic search retrieved 289 unique records. After screening the titles and abstracts, 253 records were excluded. Of the remaining 36 records, 10 were excluded after full-text review. No additional studies were added after manually screening the reference lists and searching Google Scholar. Data were extracted from 26 studies.^27^, ^28^, ^29^, ^30^, ^31^, ^32^, ^33^, ^34^, ^35^, ^36^, ^37^, ^38^, ^39^, ^40^, ^41^, ^42^, ^43^, ^44^, ^45^, ^46^, ^47^, ^48^, ^49^, ^50^, ^51^, ^52^ The detailed study selection process is summarised in the PRISMA flowchart shown in Figure 1.

### Study characteristics

Table 2 outlines the characteristics of the 26 studies included in this analysis. These studies will be published between 2019 and 2025. The countries where these studies were conducted include China (13 studies),^28^, ^29^, ^30^, ^31^, ^32^^,^^36^^,^^39^^,^^41^^,^^44^^,^^48^^,^^49^^,^^51^^,^^52^ India (3 studies),^40^^,^^42^^,^^43^ the USA (2 studies),^37^^,^^38^ the UK (1 study),^50^ Iraq (1 study),^47^ Japan (2 studies),^34^^,^^35^ Korea (1 study),^46^ Pakistan (1 study),^45^ Saudi Arabia (1 study),^38^ and Turkey (1 study).^27^

**Table 2:** Characteristics of the included studies are categorised based on the image acquisition device.

Study	Country/Ethnicity	Image acquisition device	Sample Size (Age/Gender)	Data open or not	Single or multiple centre/Study design	Disease type	Views of intraoral photograph	Reference method	Annotation procedure and number of people involved
Li et al.^30^	China/NS	Professional camera	93 (age>12/NS)	No	Single/NS	Gingivitis	Frontal view	Clinical examination (BOP, negative CA level - 3mm) and radiographic examination (negative RBL)	Manual annotation, number NS
Alalharith et al.^33^	Saudi Arabia/NS	Professional camera	134 (NS/Male and Female)	No	Single/Prospective	Gingivitis	Frontal view	Clinical examination (Löe and Silness gingival index)	Manual annotation by “dentists” but number NS
Chen & Chen^36^	China/NS	Professional camera	180 (NS/NS)	No	Single/NS	Gingivitis	Frontal view	Visual examination	Manual annotation, number NS
Li et al.^31^	China/NS	Professional camera	800 (NS/NS)	No	Single/NS	Gingivitis	Frontal view	Clinical examination (BOP, negative CA level - 3mm) and radiographic examination (negative RBL)	Manual annotation, number NS
G.-H. Li et al.^29^	China/NS	Professional camera	447 (NS/NS)	No	Single/NS	Gingivitis	Frontal view	Visual examination	Manual annotation by one dentist
Chau, Li, et al.^28^	China/Chinese	Professional camera	567 (age>18/NS)	No	Single/Prospective	Gingivitis	Frontal view	Visual examination (Oral Health Assessment Tool)	Manual annotation by one dentist
Kurt-Bayrakdar et al.^27^	Turkey/NS	Professional camera	654 (age>12/NS)	No	Single/NS	Gingivitis and gingival overgrowth	Frontal view	Visual examination	Manual annotation by 3 periodontists who reviewed the labels together
Wen et al.^49^	China/NS	Professional camera	826 (ranging from under 20 to above 50/Male and Female)	No	Single/NS	Gingivitis	Frontal view	Clinical examination (modified gingival index)	Manual annotation by 3 dentists
Vaughan et al.^50^	UK/NS	Professional camera	35 (NS/NS)	No	Single/Prospective	Gingivitis	Multiple views (Frontal and buccal)	Clinical examination (erythema or hypertrophy of the gingiva around 1 tooth at least)	Manual annotation by one orthodontist
Shen et al.^39^	China/NS	Smartphone camera (Back camera)	20 (age>35/Male and Female)	No	Single/Prospective	Gingivitis	Frontal view	Visual examination	Manual annotation by one periodontist
Snider et al.^37^	USA/NS	Smartphone camera (Back camera)	232 (age>14/NS)	No	Single/Prospective	Gingivitis	Frontal view	Clinical examination (Löe and Silness gingival index,)	Manual annotation by two dentists
Askarian et al.^38^	USA/NS	Smartphone camera (Back camera)	30 (Not specified/NS)	No	Single/Prospective	Periodontal disease (NS)	Frontal view	Visual examination	Manual annotation by one dentist
Chau et al.^52^	China/NS	Smartphone camera (Back camera)	44 (age>60/NS)	No	Multiple (3 day-care community centers) /Prospective	Gingivitis	Frontal view	Visual examination (Oral Health Assessment Tool)	Manual annotation by 2 calibrated periodontists and one dentist
Shang et al.^41^	China/NS	Intraoral camera	7220 (NS/NS)	No	Single/NS	Gingivitis	Multiple views (NS)	Visual examination	Manual annotation by “dentists” but number NS
Alam et al.^40^	India/NS	Intraoral camera	60 (NS/NS)	No	Single/Cross‑sectional	Periodontal disease (NS)	Multiple views (buccal, lingual, and occlusal views)	Clinical examination (BOP, PPD) and radiographic examination (NS)	Manual annotation by two periodontists
Liu et al.^44^	China/NS	Developed a home use device	12600 (NS/NS)	No	Multiple (10 private dental clinics)/NS	Gingivitis	Multiple views (NS)	Visual examination	Semi-automatic annotation and manual screening by 20 dental disease experts
Pingali^42^	India/NS	Developed a home use device	Hundreds (NS/NS)	No	Single/NS	Gingivitis	Multiple views (NS)	Visual examination	Manual annotation, number NS
Lodha et al.^43^	India/NS	Developed a toothbrush with a camera	1009 (NS /NS)	No	Single/NS	Periodontal disease (NS)	Multiple views (NS)	Visual examination	Manual annotation, number NS
W. Li et al.^32^	China/NS	Multiple devices (Professional camera and smartphone camera)	3932 (age>14/NS)	No	Single/Retrospective	Gingivitis	Frontal view	Visual examination	Manual annotation by 3 dentists
Li et al.^48^	China/NS	Multiple devices (Professional camera and smartphone camera)	683 (Age 14 to 60/NS)	No	Single/Retrospective	Gingivitis	Multiple views (frontal, buccal, lingual, occlusal)	Clinical examination, (clinical symptoms, CA loss) and radiographic examination (loss of supporting structures)	Manual annotation, number NS
Liu et al.^51^	China/NS	NS	3,365 (NS/NS)	Open	Multiple (Collected over the internet) /Retrospective	Gingivitis	Multiple views (Not specified)	Visual examination	Semi-automatic annotation by “dentists” but number NS
Rashid et al.^45^	Pakistan/NS	NS	517 (NS/NS)	Open	Multiple (Dental clinics and dental websites)/NS	Gingivitis	Multiple views (NS)	Visual examination	Manual annotation by “dental practitioners” but number NS
Park et al.^46^	Korea/NS	NS	220 (NS/NS)	Open	Multiple (Collected over the internet) /Retrospective	Periodontal disease (NS)	Frontal view	Visual examination	Manual annotation by 2 experts on dental hygiene, and one dentist
Moriyama et al.^35^	Japan/NS	NS	2625 (NS/NS)	No	Single/NS	Periodontitis	Frontal view	Clinical examination (PPD - 6mm)	Manual annotation by a few “dentists” but number NS
Moriyama et al.^34^	Japan/NS	NS	820 (NS/NS)	No	Single/NS	Periodontitis	Frontal view	Clinical examination (PPD - 6mm)	Manual annotation by a few “dentists” but number NS

Abbreviations: BOP, bleeding on probing; CA, clinical attachment; NS, not specified; PPD, probing pocket depth; RBL, radiographic bone loss.

The included studies were categorised into 4 groups based on the image acquisition device used: Professional SLR camera (9 studies),^27^, ^28^, ^29^, ^30^, ^31^^,^^33^^,^^36^^,^^49^^,^^50^ smartphone camera (4 studies),^37^, ^38^, ^39^^,^^52^ intraoral camera (2 studies),^40^^,^^41^ and others (11 studies). Among the other studies, 2 used both a professional SLR camera and a smartphone camera,^32^^,^^48^ 2 developed a home-use sensor device,^42^^,^^44^ one developed a camera-equipped toothbrush,^43^ and 6 studies did not specify the image acquisition device.^34^^,^^35^^,^^45^, ^46^, ^47^^,^^51^

Regarding periodontal disease type, the studies were categorised as follows: 20 studies focusing on gingivitis,^27^, ^28^, ^29^, ^30^, ^31^, ^32^, ^33^^,^^37^^,^^39^^,^^41^^,^^42^^,^^44^^,^^45^^,^^47^, ^48^, ^49^, ^50^, ^51^, ^52^ 2 studies examining periodontitis,^34^^,^^35^ and 4 studies that only mentioned periodontal disease without specification.^38^^,^^40^^,^^43^^,^^46^

The sample sizes of the studies varied considerably, ranging from 20 to 12,600, with 6 studies involving more than 1000 samples. Three studies utilised publicly available image datasets: mouth and oral diseases (MOD),^45^ PKNU-PR-ML-Lab-calculus,^46^ and intelligent diagnostic data set.^51^ Regarding the reference method, 10 studies employed clinical examinations,^30^^,^^31^^,^^33^, ^34^, ^35^^,^^37^^,^^40^^,^^48^, ^49^, ^50^ and 16 studies used visual examinations,^27^, ^28^, ^29^^,^^32^^,^^36^^,^^38^^,^^39^^,^^41^, ^42^, ^43^, ^44^, ^45^, ^46^, ^47^^,^^51^^,^^52^ with only 8 reporting the involvement of multiple experts.^27^^,^^32^^,^^37^^,^^40^^,^^44^^,^^46^^,^ ^49^^,^^52^ Four studies involved periodontists for the reference and annotation procedure.^27^^,^^39^^,^^40^^,^^52^ While most of the included studies used manual annotation, only 2 studies used a combination of semi-automatic annotation followed by manual screening.^44^^,^^51^ Only 9 studies used the AI models on multiple views of intraoral photographs,^40^, ^41^, ^42^, ^43^, ^44^, ^45^^,^^48^^,^^50^^,^^51^ while the majority (n = 17) focused on the frontal view only.^27^, ^28^, ^29^, ^30^, ^31^, ^32^, ^33^, ^34^, ^35^, ^36^, ^37^, ^38^, ^39^^,^^46^^,^^47^^,^^49^^,^^52^

### Performance of AI models

Regarding the AI models, the most commonly used task was classification (n = 17, Table 3),^30^^,^^31^^,^^34^, ^35^, ^36^, ^37^, ^38^, ^39^, ^40^^,^^42^^,^^43^^,^^45^, ^46^, ^47^, ^48^, ^49^, ^50^ followed by detection (n = 4, Table 4),^27^^,^^32^^,^^33^^,^^44^ and segmentation (n = 5, Table 5).^28^^,^^29^^,^^41^^,^^51^^,^^52^ Various AI models have been used, with Customized Convolutional Neural Networks (CNNs) being the most used in 16 studies.^27^, ^28^, ^29^^,^^32^, ^33^, ^34^, ^35^^,^^41^^,^^43^, ^44^, ^45^, ^46^^,^^48^^,^^49^^,^^51^^,^^52^ Three studies used the commercial software DentalMonitoring (DentalMonitoring, Paris, France).^37^^,^^39^^,^^50^ In 13 studies, multiple image processing techniques were employed to enhance or augment images before applying AI models.^29^^,^^30^^,^^34^^,^^36^^,^^38^^,^^40^^,^^43^, ^44^, ^45^, ^46^, ^47^, ^48^, ^49^ One study used a generative adversarial network to generate additional images for model training to improve accuracy.^34^ Thirteen studies reported the performance of the selected AI model and included other controlled AI models for comparison (Tables 3, 4, and 5).^30^, ^31^, ^32^^,^^34^, ^35^, ^36^^,^^41^^,^^43^^,^^46^, ^47^, ^48^, ^49^^,^^51^

**Table 3:** A summary of studies using AI models for classification tasks.

Study	Image preprocessing	AI model	Controlled model	Train/Valid/Test	Accuracy	Sensitivity	Specificity	F1	Precision
Askarian et al.^38^	Colour tuning and colour correction	SVM	NA	70/30/0	0.94	0.93	0.93	NA	NA
Li et al.^30^	Image enhancement	CLAHE+GLCM+ELM	NBC, WE, GLCM+ELM	73/20/0	0.74	0.75	0.73	NA	0.74
Pingali^42^	NA	Google Cloud's AutoML vision	NA	NA	0.80	False Positive 0.24	False Negative 0.14	NA	NA
Moriyama et al.^35^	NA	Customized CNN	CNN	5-fold cross validation	0.77	NA	NA	NA	NA
Moriyama et al.^34^	GAN to generate more images	Customized CNN	Without GAN	5-fold cross validation	0.85	0.74	0.90	NA	NA
Chen & Chen^36^	Adjust the size and the gray level	GLCM+ANN	BP, SA	10-fold cross-validation	0.75	0.75	0.75	0.75	0.76
Li et al.^31^	N/A	MGLCM+PSONN	BP, GA, MAGA, SA	10-fold cross-validation	0.78	0.78	0.78	0.78	0.78
Khaleel & Aziz^47^	Resize	Bat swarm	SOM, FSOM	65/55/0	0.98	NA	NA	NA	NA
Shen et al.^39^	N/A	DentalMonitoring	NA	External test (20)	Cohen's Kappa: 80% compare with periodontist	NA	NA	NA	NA
Lodha et al.^43^	Rotations, flips, and zooms	Customized CNN (MobileNet)	VGG 16, DenseNet, and VGG19	70/10/20	0.88	NA	NA	0.87	0.92
Park et al.^46^	Resize, Color normalisation	Customized CNN (Parallel 1D conv +shortcut)	ResNet152	10-fold cross- validation	0.88	NA	NA	NA	NA
Alam et al.^40^	Image resizing and color correction	NA	NA	NA	0.88	0.91	0.86	NA	NA
Li et al.^48^	Resize, normalisation, augmentation	Customized CNN (ResNet)	GoogLeNet, AlexNet, VGG	5-fold cross-validation	0.87	NA	NA	0.92	0.97
Rashid et al.^45^	Scaling, Rotation, Zooming, Horizontal flipping, Shear	Customized CNN (InceptionResNetV2)	NA	60/20/20	1.00	NA	NA	0.99	0.99
Snider et al.^37^	N/A	DentalMonitoring	NA	External test (232)	0.48	0.35	0.96	NA	NA
Wen et al.^49^	Normalisation, denoising, resizing	Customized CNN (DenseNet)	ResNet, Inception-v3, EfficientNet	5-fold cross validation	0.74	0.82	0.69	NA	NA
Vaughan, et al.^50^	NA	DentalMonitoring	NA	External test (35)	Cohen's Kappa: 0.01	1	0.09	NA	NA

Abbreviations: ANN, Artificial Neural Network; BP, back-propagation; CLAHE, contrast-limited adaptive histogram equalisation; CNN, Convolutional Neural Network; DenseNet, Dense Convolutional Network; ELM, extreme learning machine; F1, F1 score; FSOM, Fuzzy Self-Organising Map; GA, genetic algorithm; GAN, generative adversarial network; GLCM, gray-level co-occurrence matrix; MAGA, multi-agent GA; MGLCM, multichannel GLCM; NA, Not Available; NBC, naïve Bayesian classifier; Parallel 1D conv +shortcut, one-dimensional convolutions and shortcuts; PSONN, particle swarm optimisation neural network; ResNet, Residual Networks; SA, simulated annealing; SOM, Self-Organising Map algorithm; SVM, support vector machine; VGG, Visual Geometry Group; WN, wavelet energy.

**Table 4:** A summary of studies using AI models for detection tasks.

Study	Image preprocessing	AI model	Controlled model	Train/Valid/Test	Accuracy	Sensitivity	Specificity	F1	AUROC	Precision
Liu et al.^44^	Image Enhancement	Customized CNN (Customized MASK R-CNN)	NA	80/20 42 images (External test)	Recognition rate 0.94	0.97	0.95	NA	NA	NA
Alalharith et al.^33^	NA	Customized CNN (Faster R-CNN)	NA	70/30/0	0.78	NA	NA	NA	NA	0.87
W. Li et al.^32^	NA	Customized CNN	SSD, FNet+LNet	54/15/30	NA	0.67	NA	NA	0.58	NA
Kurt-Bayrakdar et al.^27^	NA	Customized CNN (YOLO v5x)	NA	80/10/10	GI: 0.64; GO: 0.56	GO: 0.76, GI: 074	NA	GO: 0.71, GI: 0.78	GO: 0.77, GI: 0.80	GO: 0.68, GI: 0.82

Abbreviations: AUROC, Area Under the Receiver Operating Characteristic Curve; CNN, Convolutional Neural Network; FNet, Mixing Tokens with Fourier Transforms; GI, Gingivitis; GO, Gingival Overgrowth; LNet, Lustre Networking Module; NA, Not Available; SSD, Single Shot Detector; YOLO, You Only Look Once.

**Table 5:** A summary of studies using AI models for segmentation tasks.

Study	Image preprocessing	AI model	Controlled model	Train/Valid/Test	Sensitivity	Specificity	AUROC	IoU	Precision
G.-H. Li et al.^29^	Random Crop, Random Rotation, Vertical Flip	Customized CNN(DeepLabv3+)	NA	337/110/0	NA	NA	0.70	0.65 (Mean)	0.61
Shang et al.^41^	NA	Customized CNN (U-Net)	PSPNet, DeepLabV3	90/10/0	NA	NA	NA	0.43 (Gingivitis specific area)	NA
Chau, Li, et al.^28^	NA	Customized CNN (DeepLabv3+)	NA	80/20/0	0.92	0.94	NA	0.60 (Mean)	NA
Liu et al.^51^	NA	Customized CNN (Oral-Mamba)	U-Net	60/20/20	NA	NA	NA	0.70	0.82
Chau, et al.^52^	NA	Customized CNN (DeepLabv3+)	NA	External test (20)	0.93	0.50	NA	NA	NA

Abbreviations: AUROC, Area Under the Receiver Operating Characteristic Curve; CNN, Convolutional Neural Network; IoU, Intersection over Union; NA, not available; PSPNet, Pyramid Scene Parsing Network.

Nearly half of the studies (11/26) used other devices or did not specify the devices for image acquisition for different tasks such as classification, detection, and segmentation.^32^^,^^34^^,^^35^^,^^42^, ^43^, ^44^, ^45^, ^46^, ^47^, ^48^^,^^51^ Therefore, the performance of AI between different image acquisition devices has not been compared directly. The performance was analysed according to individual tasks (classification, detection, and segmentation).

Out of 17 included studies using classification, 15 studies reported accuracy.^30^^,^^31^^,^^34^, ^35^, ^36^, ^37^, ^38^^,^^40^^,^^42^^,^^43^^,^^45^, ^46^, ^47^, ^48^, ^49^ Other outcome measures included sensitivity, specificity, F1-score, or precision (Table 3). For the classification task, the accuracy ranged from 0.48 to 1.00. DentalMonitoring has been tested in 3 studies.^37^^,^^39^^,^^50^ One study achieved a Cohen's Kappa of 0.80 when compared with a periodontist; a second study demonstrated an accuracy of 0.48, with a sensitivity of 0.35 and specificity of 0.96 when compared with clinical examination; and a third study showed a Cohen's Kappa of 0.01, with a sensitivity of 1.00 and specificity of 0.09 when compared to an orthodontist.^37^^,^^39^^,^^50^

Among detection (n = 4) and segmentation (n = 5) studies, outcome measures were more heterogeneous. In addition to the parameters that are used in classification studies (accuracy, sensitivity, specificity, F1-score, and precision), the area under the receiver operating characteristic curve (AUROC) and IoU were also employed (Tables 4 and 5). In the detection task, accuracy ranged from 0.56 to 0.78, and AUROC ranged from 0.58 to 0.80 (Table 4).^27^^,^^32^^,^^33^^,^^44^ For the segmentation task, 2 studies used mean IoU, which is the average of all regions of interest (ROI), ranging from 0.60 to 0.65.^28^^,^^29^ Two studies used the specific IoU for gingivitis, which was 0.43 and 0.7, respectively.^41^^,^^51^ The same model (Customized CNN DeepLabv3+) was used in 2 studies: one internal validation achieved a sensitivity of 0.92 and specificity of 0.94, while the external test study showed a sensitivity of 0.93 and specificity of 0.50.^28^^,^^52^

### Risk of bias and applicability

**Fig. 2:**
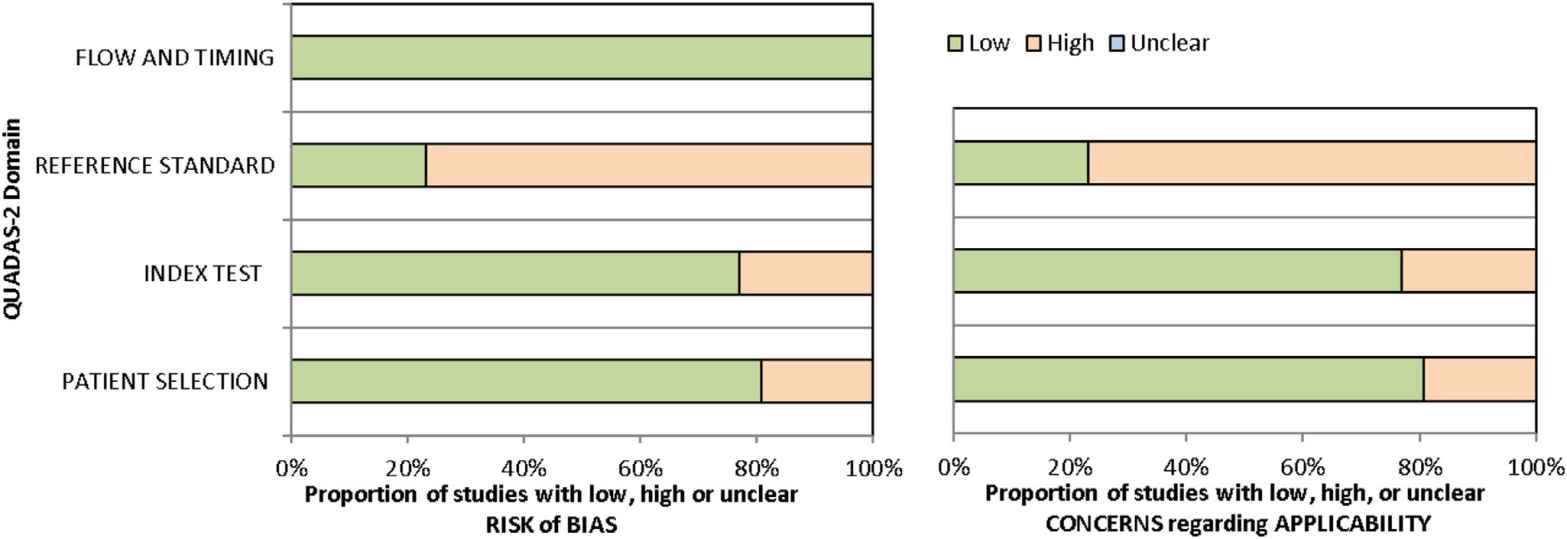
The proportion of studies with low, high, and unclear risk of bias and applicability concerns.

Figure 2 and Supplementary Table S1 provide detailed information on the risk of bias and applicability concerns. Regarding the risk of bias, 6 out of 26 included studies were deemed low across all 4 domains of QUADAS-2.^30^^,^^31^^,^^33^^,^^37^^,^^40^^,^^49^ For applicability concerns, only 6 studies were classified as having low concern in all 3 domains.^30^^,^^31^^,^^33^^,^^37^^,^^40^^,^^49^

## Discussion

This systematic review assesses the methodological characteristics and clinical performance of AI models for detecting periodontal disease using intraoral photographs. To the best of our knowledge, this is the first review to investigate the use of AI in this context, considering various image acquisition devices and AI tasks. The hypothesis that AI models using intraoral photographs achieve accuracy comparable to (80% or above) that of reference methods is rejected. This result is consistent with findings from previous reviews on AI-based periodontal disease diagnosis using non-photographic imaging modalities^53^, ^54^, ^55^ and AI models for periodontology applications remain in development. However, the clinical performance of current AI models demonstrates the feasibility of detecting periodontal disease from intraoral photographs, highlighting their potential clinical utility.

### Performance interpretation

Fifteen studies reported accuracy exceeding 0.7.^30^^,^^31^^,^^33^, ^34^, ^35^, ^36^^,^^38^^,^^40^^,^^42^^,^^43^^,^^45^, ^46^, ^47^, ^48^, ^49^ However, caution is needed when interpreting “high accuracy,” as strong performance metrics do not necessarily translate to clinical usefulness.^56^ A significant limitation across the included studies was poor reporting quality, which affected the reliable evaluation of performance metrics. Two studies reported nearly perfect (approaching 100%) single performance metrics.^46^^,^^47^ However, using just one metric can be misleading when the dataset is imbalanced, meaning one label is much more common than the others. In clinical settings, it is important to conduct a comprehensive evaluation that includes both prevalence-dependent metrics and prevalence-independent metrics.^57^, ^58^, ^59^ The lack of standardised evaluation methods and the use of unreliable metrics for assessing model performance threaten the quality and reliability of dental AI systems.^60^ To address these issues, future AI studies should improve their reporting and evaluation quality by following established guidelines, including the Dental AI Checklist,^61^ Core Outcomes Measures in Dental Computer Vision Studies (DentalCOMS),^62^ the Checklist for Artificial Intelligence in Medical Imaging (CLAIM),^63^ and “Towards a Guideline for Evaluation Metrics in Medical Image Segmentation.”^60^

AI models typically perform better on training data but show reduced accuracy on external test data due to data heterogeneity, particularly variations in image acquisition devices.^64^ This pattern was also observed in the included studies. Three studies that externally tested the same AI model (DentalMonitoring) yielded inconsistent results: one demonstrated high performance, while the other 2 indicated low accuracy.^37^^,^^39^^,^^50^ Similarly, when testing the Customized CNN DeepLabv3+ model, internal validation achieved high sensitivity (0.92) and specificity (0.94), but external testing showed a marked drop in specificity to 0.50 despite maintaining high sensitivity (0.93).^28^^,^^52^ While the samples came from different populations, this discrepancy raises concerns about clinical usefulness.

Given these common performance drops in external testing, future studies should prioritise both internal and external tests, including tests on third-party public datasets.^65^ When external tests yield poor results, cross-center training may help improve the model's usefulness. Additionally, no studies have clearly shown the inclusion of different ethnicities in the AI model development. Further investigation is needed to evaluate how variations in imaging devices and differences in patient ethnicities affect AI model performance. These factors may significantly impact clinical outcomes, affecting the reliability and applicability of AI-driven diagnostics.^66^ A deeper understanding of these effects is essential to ensuring equitable AI applications in dentistry and optimising diagnostic accuracy across diverse populations.

### Dataset challenges

Data quality is the primary concern when evaluating the methodological characteristics of included studies. It forms the foundation of a clinically useful AI model and determines its expected performance and clinical applicability.^67^ Several guidelines, such as the METRIC framework, have been proposed for systematically assessing training datasets, establishing reference datasets, and designing test datasets.^67^ Data quality can be assessed from both technical and clinical perspectives.

From a technical standpoint, poor image quality was the main issue in the 3 studies that made their datasets available.^45^^,^^46^^,^^51^ Specifically, these studies suffered from inadequate lighting, low resolution, and poor focus—issues that can lead to misinterpretation when detecting periodontal disease. A high-quality intraoral photograph should convey information about the disease location, size, colour, texture, and depth. Several established guidelines can help ensure optimal photo quality.^68^, ^69^, ^70^, ^71^

From a clinical perspective, a major limitation of the included studies was that the reference methods were not true gold standards and varied widely across studies, potentially affecting accuracy and introducing bias. The 2018 Periodontal Classification is the latest classification scheme for diagnosing health, gingivitis, and periodontitis.^72^, ^73^, ^74^, ^75^, ^76^ While probing depth is the primary clinical parameter for categorising disease types, a proper diagnosis also requires assessing bleeding on probing, radiographic bone loss, clinical attachment loss, and periodontal treatment history.^73^ Despite all studies being published after 2018, none reported their reference methods in detail according to the 2018 Periodontal Classification. Most studies focused on gingivitis due to the difficulty of diagnosing periodontitis from photographs. Only 2 studies addressed periodontitis and specified probing depth values—categorising “Healthy” as 2 mm and “Severe periodontitis” as 6 mm or more—which do not align with diagnostic gold standards.^34^^,^^35^

Clinician performance is another critical factor affecting reference methods. Using a single clinician for reference can introduce bias, as an AI model's accuracy is inherently limited by the clinician's diagnostic ability. Multiple experts, while providing more robust assessments, naturally introduce variations in their evaluations. Studies should detail their annotator validation methods, calibration processes, and approaches to addressing inter-annotator agreement. Future research must develop strategies for establishing more reliable reference methods that can serve as true “gold standards.”

### Image acquisition devices and user interaction

A wide range of image-acquisition devices have been used in the included studies, with 3 studies developing home-use devices for detecting periodontal disease.^42^, ^43^, ^44^ While home-use devices can capture multiple views of gingiva images, which may provide a more comprehensive check than smartphones, their availability and inherent cost may be prohibitive for widespread community use. In contrast, smartphones' widespread availability and cost-effectiveness make them promising tools for tele-dentistry and dental public health surveys. Further research should evaluate and compare AI model performance across different devices. Additionally, the impact of AI on patient interaction—both through software and hardware—requires careful consideration. Making AI systems more user-friendly, particularly for older populations, remains crucial.

### AI models and explainable AI

Various deep learning models have been used in the included studies, with the CNN being the most utilised. However, no consensus exists on which model performs better across different studies. In today's fast-paced AI landscape, many previous methods have become outdated and cannot compete with current state-of-the-art models. Only 5 studies evaluated segmentation models, while the others focused on classification or detection.^28^^,^^29^^,^^41^^,^^51^^,^^52^ The high cost and difficulty of obtaining pixel-wise expert annotations and the challenge for AI to achieve high IoU in segmentation may explain this. For the included studies, all IoU scores were below 0.75.

Additionally, AI is often described as a “black box” because its internal decision-making processes are not easily understandable to humans.^77^^,^^78^ Recent efforts have focused on training clinical AI to reason like a team of doctors.^79^ Researchers have developed approaches to explainable AI (XAI) that can encode this conceptual reasoning and support decisions.^79^ Concept bottleneck models (CBMs) are a promising example. These models provide both an overall prediction and a set of understandable concepts learned from data that justify model recommendations and support debate among decision-makers.^79^, ^80^, ^81^ Future research can adapt XAI for detecting periodontal disease to promote trust and confidence in its clinical use.

### Openness and ethical considerations

The openness of AI research, achieved through both open data and open-source models, is crucial to ensuring transparency and reproducibility. Providing unrestricted access to datasets and sharing source code is key to fostering innovation and ensuring rigorous validation. Of the 26 included studies in this review, only 3 provide open data. Therefore, future studies should prioritise making datasets, software, code, and trained models readily available to enhance research integrity and applicability.

As new technologies continue to emerge and integrate into daily clinical practice, ethical considerations must be acknowledged and addressed by all stakeholders, including healthcare providers, developers, policymakers, and patients. To ensure responsible implementation, future AI studies should improve their reporting and evaluation quality by adhering to established guidelines.^82^

### Potential for misclassification

Approximately one-third of dental patients may experience periodontal misclassification during clinical examination by human dentists.^83^ Similarly, AI models are also prone to classification errors, which may be attributed to annotation inaccuracies—errors stemming from incorrect labeling or categorisation by human experts—or algorithmic issues, which refer to computer errors in the processing and analysis of data. To enhance model robustness and real-world applicability, future research should investigate the underlying causes of AI misclassifications. Subsequent studies will aim to identify the key factors contributing to classification errors and develop strategies to minimise them.^83^, ^84^, ^85^

### Limitations of the study

This study has several limitations. Its scope is limited, as it focuses solely on photographs despite the use of other imaging modalities in previous studies. Additionally, many of the included studies originate from the engineering field, where the study design—whether prospective or retrospective—is not always explicitly defined. Similarly, demographic factors such as gender and ethnicity are not consistently reported, which limits the ability to assess potential biases in AI model performance. Furthermore, the review is restricted to peer-reviewed studies published in English, which not only potentially limits the breadth of insights but also introduces a linguistic bias. Although we initially intended to conduct a meta-analysis of accuracy outcomes, the significant heterogeneity and limited reporting quality prevented us from extracting enough details to perform this analysis.

## Conclusions

This systematic review highlights the feasibility of AI models for detecting periodontal disease using intraoral photographs, despite methodological differences and variations in performance metrics among studies. While many models report high accuracy, their clinical applicability remains limited. To ensure robust and clinically useful AI systems, future research should focus on improving reporting standards, standardising evaluation metrics, performing external tests, enhancing data quality, and using gold standards as reference methods. Additionally, efforts should emphasise promoting transparency, integrating ethical considerations, minimising misclassification, and advancing the creation of explainable and user-friendly AI models to enhance their clinical applicability and reliability.

## CRediT authorship contribution statement

**Kaijing Mao:** Conceptualization, Methodology, Software, Formal analysis, Investigation, Resources, Data curation, Writing – original draft, Writing – review & editing, Visualization. **Khaing Myat Thu:** Conceptualization, Methodology, Software, Formal analysis, Investigation, Resources, Data curation, Validation, Writing – review & editing. **Kuo Feng Hung:** Conceptualization, Writing – review & editing. **Ollie Yiru Yu:** Conceptualization, Writing – review & editing. **Richard Tai-Chiu Hsung:** Writing – review & editing, Supervision. **Walter Yu-Hang Lam:** Conceptualization, Methodology, Validation, Resources, Writing – review & editing, Supervision, Project administration.

## Conflict of interest

The authors declare that they have no known competing financial interests or personal relationships that could have appeared to influence the work reported in this paper.

## Appendix Supplementary materials

Image, application 1

## Footnotes

Supplementary material associated with this article can be found in the online version at doi:10.1016/j.identj.2025.100883.
