# Supplementary material for: Artificial Intelligence in Detecting Periodontal Disease From Intraoral Photographs: A Systematic Review
Source: Int Dent J. 2025 Jul 9;75(5):100883. doi: 10.1016/j.identj.2025.100883 (PMC12274314; doi:10.1016/j.identj.2025.100883)
Supplement: Supplementary file 1 [file mmc1.docx]

**Supplementary Table S1**: QUADAS-2 risk of bias and applicability concerns of included studies.

| **Study** | **RISK OF BIAS** | | | | **APPLICABILITY CONCERNS** | | |
| --- | --- | --- | --- | --- | --- | --- | --- |
|  | **PATIENT SELECTION** | **INDEX TEST** | **REFERENCE STANDARD** | **FLOW AND TIMING** | **PATIENT SELECTION** | **INDEX TEST** | **REFERENCE STANDARD** |
| Alalharith et al. 2020(1) | ☺ | ☺ | ☺ | ☺ | ☺ | ☺ | /☺ |
| Alam, Alanazi, Alshehri, & Chowdhury 2024(2) | ☺ | ☺ | ☺ | ☺ | ☺ | ☺ | ☺ |
| Askarian, Tabei, Tipton, & Chong 2019(3) | ☹ | ☺ | ☹ | ☺ | ☹ | ☺ | ☹ |
| Chau et al. 2023(4) | ☺ | ☹ | ☹ | ☺ | ☺ | ☹ | ☹ |
| Chau, et al. 2025 (5) | ☺ | ☹ | ☹ | ☺ | ☺ | ☹ | ☹ |
| Chen & Chen 2020(6) | ☺ | ☺ | ☹ | ☺ | ☺ | ☺ | ☹ |
| G.-H. Li et al. 2021(7) | ☺ | ☹ | ☹ | ☺ | ☺ | ☹ | ☹ |
| Khaleel & Aziz 2021(8) | ☹ | ☹ | ☹ | ☺ | ☹ | ☹ | ☹ |
| Kurt Bayrakdar et al. 2023(9) | ☺ | ☺ | ☹ | ☺ | ☺ | ☺ | ☹ |
| Li et al. 2019(10) | ☺ | ☺ | ☺ | ☺ | ☺ | ☺ | ☺ |
| Li et al. 2020(11) | ☺ | ☺ | ☺ | ☺ | ☺ | ☺ | ☺ |
| Li et al. 2024 (12) | ☺ | ☺ | ☹ | ☺ | ☺ | ☺ | ☹ |
| Liu et al. 2019(13) | ☺ | ☺ | ☹ | ☺ | ☺ | ☺ | ☹ |
| Liu et al. 2024 (14) | ☹ | ☹ | ☹ | ☹ | ☹ | ☹ | ☹ |
| Lodha et al. 2023(15) | ☺ | ☺ | ☹ | ☺ | ☺ | ☺ | ☹ |
| Moriyama et al. 2019(16) | ☺ | ☺ | ☹ | ☺ | ☺ | ☺ | ☹ |
| Moriyama et al.2019(17) | ☺ | ☺ | ☹ | ☺ | ☺ | ☺ | ☹ |
| Park et al. 2023(18) | ☹ | ☺ | ☹ | ☺ | ☹ | ☺ | ☹ |
| Pingali 2019(19) | ☹ | ☺ | ☹ | ☺ | ☹ | ☺ | ☹ |
| Rashid et al. 2024(20) | ☺ | ☺ | ☹ | ☺ | ☺ | ☺ | ☹ |
| Shang, Li, & Li 2021(21) | ☺ | ☺ | ☹ | ☺ | ☺ | ☺ | ☹ |
| Shen et al. 2022(22) | ☺ | ☹ | ☹ | ☺ | ☺ | ☹ | ☹ |
| Snider et al. 2024(23) | ☺ | ☺ | ☺ | ☺ | ☺ | ☺ | ☺ |
| Vaughan, et al. 2025 (24) | ☺ | ☺ | ☹ | ☺ | ☺ | ☺ | ☹ |
| W. Li et al. 2021(25) | ☺ | ☺ | ☹ | ☺ | ☺ | ☺ | ☹ |
| Wen et al. 2024 (26) | ☺ | ☺ | ☺ | ☺ | ☺ | ☺ | ☺ |

☺Low Risk ☹High Risk ? Unclear Risk

**Reference**:

1. Alalharith DM, Alharthi HM, Alghamdi WM, Alsenbel YM, Aslam N, Khan IU, et al. A deep learning-based approach for the detection of early signs of gingivitis in orthodontic patients using faster region-based convolutional neural networks. International Journal of Environmental Research and Public Health. 2020;17(22):8447.

2. Alam MK, Alanazi NH, Alshehri ADA, Chowdhury F. Accuracy of Al Algorithms in Diagnosing Periodontal Disease Using Intraoral Images. Journal of Pharmacy and Bioallied Sciences. 2024;16(Suppl 1):S583-S5.

3. Askarian B, Tabei F, Tipton GA, Chong JW, editors. Smartphone-based method for detecting periodontal disease. 2019 IEEE Healthcare Innovations and Point of Care Technologies,(HI-POCT); 2019: IEEE.

4. Chau RCW, Li G-H, Tew IM, Thu KM, McGrath C, Lo W-L, et al. Accuracy of artificial intelligence-based photographic detection of gingivitis. international dental journal. 2023;73(5):724-30.

5. Chau RCW, Cheng ACC, Mao K, Thu KM, Ling Z, Tew IM, et al. External Validation of an AI mHealth Tool for Gingivitis Detection among Older Adults at Daycare Centers: A Pilot Study. International Dental Journal. 2025.

6. Chen Y, Chen X, editors. Gingivitis identification via GLCM and artificial neural network. Medical Imaging and Computer-Aided Diagnosis: Proceeding of 2020 International Conference on Medical Imaging and Computer-Aided Diagnosis (MICAD 2020); 2020: Springer.

7. Li G-H, Hsung T-C, Ling W-K, Lam WY-H, Pelekos G, McGrath C, editors. Automatic site-specific multiple level gum disease detection based on deep neural network. 2021 15th International Symposium on Medical Information and Communication Technology (ISMICT); 2021: IEEE.

8. Khaleel BI, Aziz MS, editors. Using artificial intelligence methods for diagnosis of gingivitis diseases. Journal of Physics: Conference Series; 2021: IOP Publishing.

9. Kurt Bayrakdar S, Uğurlu M, Yavuz MB, Sali N, Bayrakdar İŞ, Çelik Ö, et al. Detection of tooth numbering, frenulum attachment, gingival overgrowth, and gingival inflammation signs on dental photographs using convolutional neural network algorithms: a retrospective study. 2023.

10. Li W, Chen Y, Sun W, Brown M, Zhang X, Wang S, et al. A gingivitis identification method based on contrast‐limited adaptive histogram equalization, gray‐level co‐occurrence matrix, and extreme learning machine. International Journal of Imaging Systems and Technology. 2019;29(1):77-82.

11. Li W, Jiang X, Sun W, Wang SH, Liu C, Zhang X, et al. Gingivitis identification via multichannel gray‐level co‐occurrence matrix and particle swarm optimization neural network. International Journal of Imaging Systems and Technology. 2020;30(2):401-11.

12. Li W, Guo E, Zhao H, Li Y, Miao L, Liu C, et al. Evaluation of transfer ensemble learning-based convolutional neural network models for the identification of chronic gingivitis from oral photographs. BMC Oral Health. 2024;24(1):814.

13. Liu L, Xu J, Huan Y, Zou Z, Yeh S-C, Zheng L-R. A smart dental health-IoT platform based on intelligent hardware, deep learning, and mobile terminal. IEEE journal of biomedical and health informatics. 2019;24(3):898-906.

14. Liu Y, Cheng Y, Song Y, Cai D, Zhang N. Oral screening of dental calculus, gingivitis and dental caries through segmentation on intraoral photographic images using deep learning. BMC Oral Health. 2024;24(1):1287.

15. Lodha N, Pal A, Das S, Roy S, Chakraborty S, Pandey SK, editors. Deep Learning Empowered IoT Toothbrush: A Paradigm Shift in Dental Health Monitoring. 2023 International Conference on Artificial Intelligence for Innovations in Healthcare Industries (ICAIIHI); 2023: IEEE.

16. Moriyama Y, Lee C, Date S, Kashiwagi Y, Narukawa Y, Nozaki K, et al., editors. A MapReduce-like Deep Learning Model for the Depth Estimation of Periodontal Pockets. HEALTHINF; 2019.

17. Moriyama Y, Lee C, Date S, Kashiwagi Y, Narukawa Y, Nozaki K, et al., editors. Evaluation of dental image augmentation for the severity assessment of periodontal disease. 2019 International Conference on Computational Science and Computational Intelligence (CSCI); 2019: IEEE.

18. Park S, Erkinov H, Hasan MAM, Nam S-H, Kim Y-R, Shin J, et al. Periodontal disease classification with color teeth images using convolutional neural networks. Electronics. 2023;12(7):1518.

19. Pingali L, editor Personal oral health advisor using multimodal sensing and machine learning with smartphones and cloud computing. 2019 IEEE International Conference on Cloud Computing in Emerging Markets (CCEM); 2019: IEEE.

20. Rashid J, Qaisar BS, Faheem M, Akram A, Amin Ru, Hamid M. Mouth and oral disease classification using InceptionResNetV2 method. Multimedia Tools and Applications. 2024;83(11):33903-21.

21. Shang W, Li Z, Li Y, editors. Identification of common oral disease lesions based on U-Net. 2021 IEEE 3rd International Conference on Frontiers Technology of Information and Computer (ICFTIC); 2021: IEEE.

22. Shen KL, Huang CL, Lin YC, Du JK, Chen FL, Kabasawa Y, et al. Effects of artificial intelligence‐assisted dental monitoring intervention in patients with periodontitis: a randomized controlled trial. Journal of Clinical Periodontology. 2022;49(10):988-98.

23. Snider V, Homsi K, Kusnoto B, Atsawasuwan P, Viana G, Allareddy V, et al. Clinical evaluation of Artificial Intelligence Driven Remote Monitoring technology for assessment of patient oral hygiene during orthodontic treatment. American Journal of Orthodontics and Dentofacial Orthopedics. 2024;165(5):586-92.

24. Vaughan M, Mheissen S, Cobourne M, Ahmed F. Diagnostic accuracy of artificial intelligence for dental and occlusal parameters using standardized clinical photographs. American Journal of Orthodontics and Dentofacial Orthopedics. 2025.

25. Li W, Liang Y, Zhang X, Liu C, He L, Miao L, et al. A deep learning approach to automatic gingivitis screening based on classification and localization in RGB photos. Scientific Reports. 2021;11(1):16831.

26. Wen C, Bai X, Yang J, Li S, Wang X, Yang D. Deep learning based approach: automated gingival inflammation grading model using gingival removal strategy. Scientific Reports. 2024;14(1):19780.
